# Using SmartQuit®, an Acceptance and Commitment Therapy Smartphone application, to reduce smoking intake

**DOI:** 10.1177/2055207617729535

**Published:** 2017-09-19

**Authors:** Satvir Singh, Nicola J Starkey, Rebecca J Sargisson

**Affiliations:** School of Psychology, 3717 University of Waikato, New Zealand

**Keywords:** Smoking, cigarettes, cravings, Acceptance and Commitment Therapy, SmartQuit®, New Zealand, behaviour

## Abstract

**Objective:**

SmartQuit® is a smartphone application (app) for smoking cessation based on Acceptance and Commitment Therapy, a behavioural therapy that encourages individuals to accept internal experiences, such as cravings to smoke, without acting on those experiences or urges. We used a single-subject (A-B-A) design with 10 participants to examine whether SmartQuit® use would reduce cigarette intake in a New Zealand sample.

**Methods:**

10 smokers tallied their own cravings experienced and cigarettes smoked then sent those tallies to the first author every day until we observed stable patterns (Phase A1). We then gave the participants individual access to the SmartQuit® app (Phase B). When they advised that they had ceased using the app, they again recorded daily cravings and cigarettes smoked for a minimum of three days (Phase A2). We also collected follow-up smoking and craving data at 1, 2 and up to 13 months after completion of Phase A2.

**Results:**

Using SmartQuit® reduced our participants’ daily cigarette intake significantly in the short-term and three individuals remained smoke-free up to 13 months later. Cravings to smoke did not differ significantly across Phases A1, B and A2, but graphical analysis showed a trend for decreasing cravings.

**Conclusion:**

Our results suggest that SmartQuit® provides another readily accessible intervention to help people stop smoking and is suited for use with a New Zealand population.

## Introduction

While smoking rates have declined since the 1980s, with large reductions recorded between 1980 and 2012,^1^ smoking remains a major cause of premature death,^2^ causing 9% of deaths worldwide and 18% in high-income countries.^3^ In New Zealand, tobacco use peaked in the 1970s and then dropped from approximately 35%^4^ to 17% of the New Zealand adult population in 2014.^5^ The highest smoking rates are for people of Māori ethnicity with 38% of adult Māori reporting being smokers.^5^

It is important to keep reducing the number of people who smoke and many smokers do try to quit. For example, between 60% and 85% of smokers in a large survey of American households reported that they had made a serious attempt to quit smoking in the previous year.^6^ However, of those who attempted to quit only 5–9% were not smoking six months later,^6^ showing that quitting smoking is difficult.

The availability of mobile health (mHealth) interventions has increased over the last few years partly due to their potential to provide access to health services for hard-to-reach populations and often at a fraction of the cost of face-to-face service delivery. While most mobile phone smoking cessation interventions thus far have used text messaging as a central component,^7,8^ with the spread of smartphones able to run computer programs or applications,^7^ there are new possibilities for using mobile phones to reduce smoking intake. However, according to an extensive recent review of research on mobile phone interventions for smoking cessation,^8^ research on smartphone application-based interventions is scarce.

Abroms et al. ^7^ suggest that health-based applications (apps) could be improved through integration with evidence-based practices, such as behavioural interventions. Acceptance and Commitment Therapy (ACT) is a recent behaviour therapy that has grown out of the behavioural tradition.^9^ The foundation of ACT is Relational Frame Theory, which offers a theoretical account of human language and cognition. The primary therapeutic goal of ACT is to increase psychological flexibility – defined as contacting the present moment – and preserving or varying behaviour to achieve valued goals. These goals are achieved through decreasing experiential avoidance, the process of suppressing and avoiding unwanted feelings, sensations, thoughts, and other internal events.^10^ Under an ACT framework, clients are taught not to fight or avoid challenges but instead to embrace and accept them whilst acting on their fundamental values.^11^ Being aware or mindful of urges to smoke is preferred over symptom reduction, as the aim is to change the person’s relationship with their symptoms.^12^

With reference to smoking cessation, an ACT approach would be to support the client with identifying internal and external cues associated with smoking; to learn strategies to manage these triggers;^13^ and to view cravings to smoke as mere thoughts rather than as reasons to smoke cigarettes.^14^ In an ACT programme, clients are encouraged to commit to their identified values such as caring for one’s body and health,^14^ and to act on those values, for example, by choosing a quit date and selecting a method to quit smoking.^14^ To summarise, the ‘acceptance’ component of ACT helps individuals to identify and accept inner triggers to smoke and not to avoid withdrawal symptoms. The ‘commitment’ component emphasises the articulation of personal values and committing to stop smoking.^15^

ACT has shown promise for the treatment of depression, anxiety, substance abuse, psychosis, stress and chronic pain.^14^ While these problems differ in their functions, they all involve individuals who try to reduce or control private aversive events. Evidence suggests that while the attempt to avoid internal cues such as those to smoke may be effective in the short term, it is not an effective long-term strategy.^14^

Researchers have found support for the use of ACT for smoking cessation. ACT combined with smoking cessation medication resulted in higher short- and long-term quit rates than the medication alone.^16^ Hernández-López et al.^14^ found that ACT, delivered by a trained ACT therapist, was just as efficacious but more cost-effective than Cognitive Behavioural Therapy (CBT) as a treatment for smoking cessation. Participants in an ACT condition experienced similar treatment effects but a five times higher abstinence rate compared to participants in a CBT condition, who experienced higher relapse rates.^14^ ACT smoking cessation treatment also produces better long-term outcomes compared to Nicotine Replacement Therapy.^13^

The ACT component of the intervention – rather than the manner in which the treatment is delivered – appears to be the key factor in treatment success. Bricker et al.^17^ found that a web-based intervention for smoking cessation based on ACT called WebQuit® was more successful than a web-based intervention that did not incorporate ACT principles. Participants in the WebQuit® condition engaged with the app more often and were more satisfied than those using the non-ACT intervention. Furthermore, at a three-month follow-up, 23% of the individuals in the WebQuit® condition had not smoked for at least 30 days compared to 10% for those in the non-ACT group.

SmartQuit® is a relatively new mobile phone application based on ACT principles. The underlying goal of SmartQuit® is to teach individuals techniques to accept their urges and not act on those urges to smoke. SmartQuit® first asks users to complete a quit plan, which contains many personal questions in order to provide an understanding of the user’s behaviours and attitudes toward smoking, along with their stress levels and the amount of money spent on smoking. The quit plan includes a proposed quit date, which can be reset. Following completion of the quit plan – which can be updated at any time – the user begins to record their urges to smoke on the app by tapping the screen of their device, to complete daily exercises by swiping the screen of their device to open the exercise and to access the available resources. There are eight short exercises to complete and the app recommends that these be completed more than once. The exercises guide the user through tasks involving learning to accept urges and act on values that guide the user towards quitting smoking. For example, the exercise might ask the user to imagine their craving to smoke as a monster who is pulling a rope. The user is advised to let go of the rope rather than to resist the craving by tugging on the rope. Each time one exercise is completed, another is unlocked. For every 10 urges recorded but not acted on, and for every exercise completed, a badge is earned. The app also includes a section for anytime coaching which includes stories from other users, tips on managing urges, an ‘ask a coach’ section and many more features.

There is some evidence from Bricker et al.^18,19^ and Zeng et al.^20^ that SmartQuit® is a useful tool. Bricker et al.^18^ compared SmartQuit® with QuitGuide®, a non-ACT smoking-cessation app that follows US clinical practice guidelines. Individuals using SmartQuit® accessed the app at significantly higher rates and rates for quitting were higher (15% vs 8%) than for QuitGuide®. However, to date, there have been no independent evaluations of SmartQuit®. Additionally, to our knowledge, there has been no evaluation of the app conducted outside the United States.

Given the high rates of smoking in New Zealand, and the particularly high rates among Māori, it is important to identify interventions that reduce smoking intake. Most New Zealand adults have access to a smartphone (71% of 18- to 54-year-olds in 2013),^21^ so treatments based on smartphone applications have the potential to reach large numbers of New Zealand smokers. Such treatments are low-cost, which makes them accessible to low-income populations. Additionally, the privacy, accessibility and anonymity of smartphone smoking cessation apps may appeal to some smokers.

We aimed to investigate whether SmartQuit® use was able to reduce the number of cigarettes smoked by a sample of New Zealand smokers. We used a small-*N* (sample size), within-subjects, design which has many benefits^22^ and was used in this study for several reasons. First, this design ensures that all participants receive the intervention – there is no control condition – as each participant acts as their own control by providing pre-intervention data on smoking and craving rates. Second, using the participants as their own control reduces error variability. Third, single-subject design involves repeated measures at baseline and post-intervention, which is difficult to achieve in some research contexts but is facilitated by the use of technology.^23^ Participants in our study were able to report cigarette consumption and cravings in real time whilst using the app which improved the validity of our measurements. Additionally, during SmartQuit® use both the data collection and the intervention was digital and remote. This meant that we were able to minimise external contact with the participants during this time so improving treatment fidelity.^23^ Fourth, analysing the outcome from use of the app though graphical analysis allowed us to assess whether the app produced a noticeable, practical reduction in the smoking rates of individual participants, and to observe the changes in behaviour over time. Between-subject designs typically measure behaviour at distinct time points, such as before and after engagement with an intervention; therefore, they provide little information about changes in behaviour over the course of the treatment.^23^ Finally, this design allowed for flexibility which meant that individuals progressed through the study at their own pace.

Our study extends earlier evaluations of SmartQuit® made by Bricker et al.^18,19^ through: (a) assessing whether use of the app would decrease cigarette intake for a New Zealand sample of smokers; (b) applying a different research design; and (c) using an independent research team.

## Method

### Participants

We recruited participants who responded to fliers posted on social media, in newspapers, and in various tertiary institutions and medical centres in the Waikato and Bay of Plenty areas, New Zealand. Five male (M1–M5) and five female (F1–F5) smokers participated. Participants M1 and F1 were of Māori ethnicity, M5 was of European Indian ethnicity, and the remaining participants were of New Zealand European ethnicity. All participants were between 25- and 44-years-old. To be eligible for participation, potential participants had to be English-speakers, 18-years-old or older, had smoked 10 or more cigarettes per day over the previous 12 months, and neither seeking nor undergoing psychological treatment for any mental health issues. They had to have scored 4 or more on the Heaviness of Smoking Index (HSI);^24^ must want to quit smoking in the near future; have access to a smartphone compatible with the SmartQuit® program; and agree not to use any other interventions or medication throughout the study.

### Materials and measures

We provided participants with an information sheet, an eligibility survey (with questions described in the Participants section), a consent form and the HSI^24^ to determine eligibility for the study. The HSI is a two-item measure based on the Fagerström test for Nicotine Dependence. The summing of the two items provides a total score, with a score of 4 or higher being suggestive of high nicotine dependence. Etter^25^ reported Cronbach alpha coefficients for internal consistency of 0.63 and Borland et al.^26^ found evidence for its validity.

Eligible participants received a small paperback notebook (10 × 8 × 0.4 cm^3^) that they carried with them in which they tallied each cigarette consumed and craving experienced under a date heading. Participants texted the daily totals to the first author at the end of each day in the A1 and A2 phases.

### Design

We used a single-subject, A1-B-A2-A3-A4-A5 design. A phases were conducted in the absence of the app, B phases during app use. A2 occurred as soon as participants stopped using the app. Phases A3, A4 and A5 were follow-up phases conducted 1 (A3), 2 (A4), and 7 - 13 (A5) months after completion of Phase A2.

### Procedure

*A phases (no app).* During Phase A1, potential participants were given an information sheet and completed a demographic questionnaire and the HSI in the presence of the first author. If the participant was eligible and wished to participate, they then signed an informed consent form. The first author asked participants to continue with their normal smoking behaviour and to respond to cravings as they usually would. Each time they smoked a cigarette or experienced a craving to smoke they were to record it in the notebook provided under the day’s date. The first author sent a text message using her own phone to each participant at the end of each day during Phases A1 and A2 to request their daily counts. The text was sent between 9p.m. and 11p.m. with the timing of the text agreed upon after discussion with each participant, and aligning as closely as possible to the time the participant reported that they usually smoked their last cigarette for the day. Once intake and cravings in Phase A1 were not trending upwards or downwards, the first author advised the participant that they could now begin using the app (Phase B). Phase A2 was similar to A1 but began after participants advised that they had ceased using the app, which occurred when participants had completed all required activities and reached their quit date.

*Phase B (SmartQuit®).* The first author provided each participant with instructions to download the SmartQuit® app (Version 2.0), along with an individual login ID and password to gain access. Participants used the app as any other consumer might. The length of Phase B depended on how long it took participants to reach – and whether they reset – their quit date and varied from two weeks to six months.

During the intervention phase, we avoided contact with the participants so as not to interfere with the app. That is, we intended to measure the effectiveness of the app on its own without additional interventions. However, we did contact participants who had not accessed the app for seven consecutive days via text message or phone. We did not contact the participant more than once. If the contact was via a phone call, the first author asked how the participant was and how they were finding their app use. These opening questions led to a discussion about why they had not been engaging with the app, with the reason often being that they were happy with their progress and felt that they did not need to continue. If a text message was sent, it read:

Hi, I hope things are well with you. I'm writing this message as I have noticed on the dashboard that you have not been using the app for a period exceeding 7 days. Just wanted to check if things are going well. Thanks.

Participants recorded cravings via the app during this phase but not the number of cigarettes smoked because the core focus of the app is to notice and allow cravings to pass rather than to record the cigarettes smoked.

*Phases A3, A4 and A5 (follow-ups).* We asked participants to report the number of cigarettes they were smoking each day at 1 month (Phase A3), 2 months (Phase A4), and between 7 and 13 months (Phase A5) after the completion of Phase A2. These data are not available for all participants as we were unable to contact some participants.

## Results

**Figure 1.**
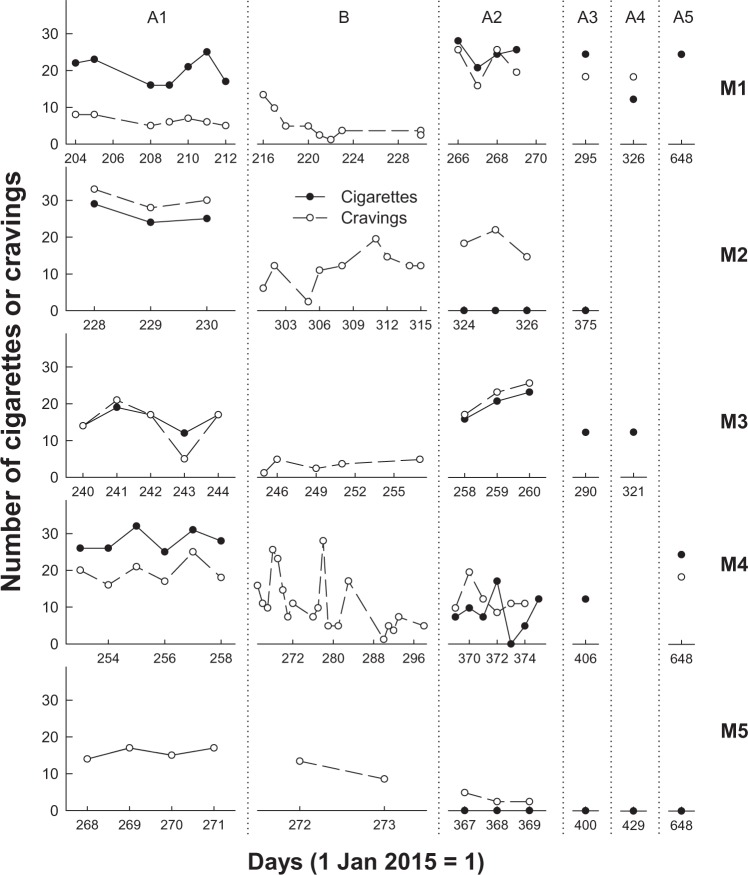

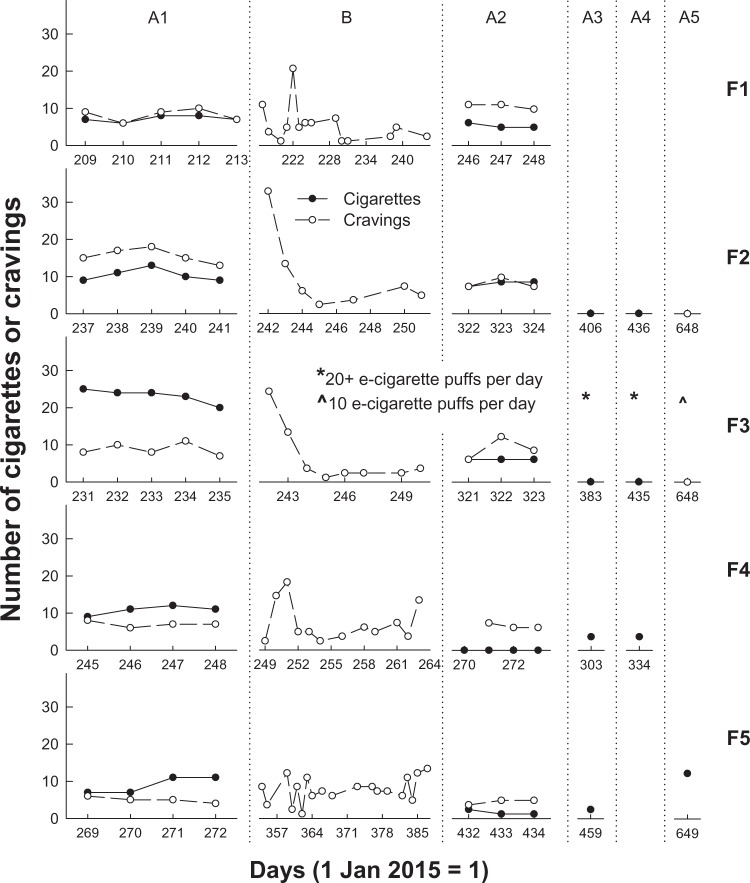
Number of cigarettes consumed (closed circles) and cravings experienced (open circles) for male (a) and female (b) participants on each day (where 1 January 2015 = 1) of recording for all phases for which data are available.

Figures 1(a) and 1(b) present the daily cigarette intake (closed circles) and cravings (open circles) for male (a) and female (b) participants during all phases for which data are available.

All participants had variable but stable levels of cigarette intake and cravings during A1. For 5 of the 10 participants, in Phase A1 intake was higher than cravings. For two participants, intake was lower than cravings and for three intake and cravings were about the same, with cravings and intake overlapping perfectly for M3. During app use in Phase B, three participants (M1, F2, and F3) experienced an increase in cravings upon initiating app use, which rapidly declined with continued app use. For most participants, however, cravings continued at similar rates as they had during Phase A1 and for some, cravings decreased through Phase B (M1, M4, F1, F2, and F3). In Phase A2, after participants had ceased using the app, cigarette intake decreased for six participants and remained similar to A1 consumption for 4 of the 10 participants (M1, M3, F1, and F2). Of the six participants whose intake decreased, three (M2, M5, and F4) reduced their intake during Phase A2 to zero. During Phase A2, cravings decreased relative to cravings in A1 for two participants (M4 and M5), increased for one participant (M1) and remained similar for the remaining seven participants.

Eight of the nine participants for whom we have Phase A3 data had maintained or reduced their cigarette intake compared to intake during Phase A1. M1’s smoking intake during Phase A3 was similar to that in both A1 and A2 phases. At the two-month follow-up (Phase A4), five of the six participants for whom we have data, all except M1, had continued to maintain reductions in cigarette intake compared to Phase A1. Data for six participants were available between 7 and 13 months after the completion of Phase A2 (Phase A5). Three of these six participants (M1, M4, and F5) were smoking at pre-intervention rates during Phase A5, but the other three were smoke-free (M5, F2, and F3). Note that F3 reported that she had switched to e-cigarettes during Phase A3, and at Phase A5 was consuming 10 e-cigarette puffs per day.

Using the number of cigarettes consumed on the last day of Phases A1, A2, and the last available A phase for all 10 participants, a repeated-measures analysis of variance (ANOVA) showed a significant effect of phase on intake, *F*(2, 16) = 9.49, *p* = .002, *r* = .74. Bonferroni pairwise comparisons showed that mean intake on the last day in Phase A1, *M* = 17.2, 95% confidence interval (CI) [12.3, 22.2], was significantly higher than on the last day in Phase A2, *M* = 7.0, 95% CI [.7, 13.3], *p* = .04, and higher than on the day of the last A phase, *M* = 6.4, 95% CI [.6, 12.3], *p* = .02. Mean intake did not differ from the last day in Phase A2 to the last A phase, *p* = 1.0. These results show that intake dropped significantly after participants completed the app and had remained low up to 13 months later.

Daily craving data were not gathered consistently after Phase A2 but a repeated-measures ANOVA using the number of daily cravings reported for the last day of Phases A1, B and A2 showed no significant difference in cravings across phases, *F*(2, 18) = 3.21, *p* = .06, *r* = .51. The mean number of cravings on the last day in each phase was not significantly different to the mean number of cravings in any other phase, *M*_A1_ = 12.5, 95% CI [6.7, 18.3]; *M*_A2_ = 5.8, 95% CI [3.2, 8.4]; *M*_ALast_ = 9.0, 95% CI [4.8, 13.2], *p* > .05. However, while the means were not significantly different across phase, there was some suggestion that cravings were less frequent after engagement with the app as the mean number of cravings were lower in A2 and at the last measurement compared to baseline (A1).

In the follow-up surveys, six participants (total current participants *n* = 8) agreed that SmartQuit® was appropriate for them, with one participant reporting being undecided and the last disagreeing. The participant who disagreed that the app was appropriate for him (M3), reported that he only accessed the app 0–1 times per week and that he ‘didn’t engage with the app’. Favourable aspects mentioned by the participants were its ease of use; the pictures and tips; the techniques to manage cravings; including ownership and accountability of urges; notifications; and acknowledging and tracking urges. Negative aspects were that there was a lack of clarity on when to complete each activity; the completion certificate did not act as a motivator; it took too long to navigate through the app; and that there was no way to track urges when driving or when their phone was not in reach. Two female participants commented on the isolation they felt in using the app, suggesting that a way to connect with other smokers using the app would be helpful. Participants stated that the best strategies learnt from SmartQuit® were how to identify urges, and how to take ownership of and manage their cravings. Suggestions for improvement included the addition of voice activation as a way to engage with the app; an incentive, such as a badge, for smoke-free days; a lock on the exercises so that exercises could only be unlocked after a day or so of practicing the previous exercise; a peer aspect to feel connected; and a diary of accountability.

## Discussion

We aimed to examine whether SmartQuit® use would decrease cigarette intake for a New Zealand sample of smokers, and overall our data showed a trend of reduced cigarette intake. 6 of the 10 participants had noticeably reduced their cigarette intake (Figure 1) after using the app (Phase A2) and, 1–2 months after using the app (Phases A3 and A4), 7 of the 10 had maintained or further reduced their intake from Phase A2. Up to 13 months after the completion of Phase A2, 3 of the 10 participants were still smoke-free. Results of statistical tests showed that intake significantly decreased after use of the app. Our results with a New Zealand sample were consistent with those of Bricker et al. ^18,19^ who, using the same version as we did (2.0) reported reductions in smoking rate for 75% of all participants and 88% who *completed* the SmartQuit® programme.

While we found reductions in cigarette intake after engaging with SmartQuit®, we found that cravings to smoke did not significantly reduce over the short-term (Phases A1, B and A2). However, there was a trend indicating that cravings were reducing over time.Figure 1 shows that prior to SmartQuit® use the number of cigarettes smoked per day was higher than the number of cravings for five participants; the same as the number of cravings for three participants; and that only two participants (M2 and F2) had a higher number of cravings than cigarettes smoked. This suggests that prior to the intervention, participants were responding to their cravings by smoking, and were smoking even in the absence of cravings. After the intervention in Phase A2, the number of daily cravings remained similar to A1 baseline rates but exceeded the number of cigarettes smoked for 7 of the 10 participants. These results suggest that SmartQuit® use did not initially result in fewer cravings to smoke, but that it enabled smokers to experience their cravings while not acting on them by smoking. For some participant data in Figures 1(a) and 1(b), there is a suggestion that cravings were beginning to fade away over time (e.g. M5, F2 and F3) and the mean number of cravings was lower (although not significantly) after engagement with the app.

The most commonly used behavioural intervention for smoking cessation is CBT,^15^ which aims to help smokers reduce or avoid internal cues to smoke such as cravings. ACT in contrast, attempts to help people to *stop* trying to control or avoid internal cues such as cravings to smoke.^15^ ACT instead encourages individuals to experience and accept their cravings,^27^ and to allow the cravings to pass without acting on them. While reducing the craving to smoke was not a necessary precursor to smoking reduction in our study, our findings suggest that the frequency of cravings is likely to taper off after engagement with an ACT-based intervention such as SmartQuit®.

Our participants provided comments in qualitative responses to our questionnaire that can inform improvements to SmartQuit® specifically, but which apply to mHealth interventions more generally. mHealth interventions could be improved by making them easier to use (e.g. including voice activation and voice-recognition options, providing clear instructions and simple navigation pathways) and reducing the isolation of mHealth interventions by including features which allow connection with other users.

A limitation of our study was the reliance on self-reported data. However, given the repeated nature of our measurements, it was not feasible to request biological samples from our participants. Evidence for the validity of self-reported smoking behaviour has been provided by other researchers.^28, 29^ The number of times per week that our participants *reported* using SmartQuit® was significantly positively correlated with the number of times the app was *actually* accessed per week, *r*_s_ = .84, *p* = .009, suggesting that our self-reported data were a fair estimate of actual behaviour.

Contacting participants and asking them to record data on their smoking behaviour may have increased the risk of social desirability biases.^30^ However, if social desirability or reactivity were affecting reports, we might expect rates of cigarette consumption and cravings to be uniformly lower (in response to social pressure against smoking or reactivity in general) or lower after the intervention (in response to an expectation that the intervention would result in lower rates of smoking and craving). If cigarette intake and craving rates were lowered overall, our ability to detect the effect of the intervention would be reduced which would not enhance our results. Additionally, not all participants reported a decrease in cigarette consumption after the intervention (M1 and M3) and 8 of the 10 participants did not report a reduction in the number of daily cravings after the intervention, suggesting that the influence of social desirability was minimal.

## Conclusions

Using a single-subject design, we demonstrated support for SmartQuit® for smoking reduction with a New Zealand sample. Single-subject, small-*N*, or A-B-A designs hold great promise for evaluating the outcomes of mHealth behavioural interventions. While randomised control trials (RCT) are the ‘gold standard’ in assessing health interventions,^31^ they are time-intensive, expensive and require large numbers of participants.^32^ Due to the rapid evolution of mHealth technologies, single-subject designs which are quicker, less expensive and involve fewer participants, have distinct advantages over RCT approaches.^31, 32^ Mobile phone apps, such as SmartQuit®, provide for frequent data collection during treatment; and the data can be used to assess change over time,^31^ treatment adherence and within-subject variability.^32^ Apps designed for use on mobile devices have great potential for smoking reduction specifically and health generally as access to such devices is widespread and increasing.
